# Epidermoid cyst of the cecum resected by single-incision laparoscopic colectomy: a case report

**DOI:** 10.1186/s40792-021-01138-2

**Published:** 2021-02-25

**Authors:** Tetsuro Tominaga, Takashi Nonaka, Akiko Fukuda, Masaaki Moriyama, Shosaburo Oyama, Mitsuhisa Ishii, Terumitsu Sawai, Nozomi Ueki, Takeshi Nagayasu

**Affiliations:** 1 Departments of Surgical Oncology, Nagasaki University Graduate School of Biomedical Science, 1-7-1 Sakamoto, Nagasaki, 852-8501 Japan; 2 Departments of Cardiopulmonary Rehabilitation Science, Nagasaki University Graduate School of Biomedical Science, 1-7-1 Sakamoto, Nagasaki, 852-8501 Japan; 3 Department of Tumor and Diagnostic Pathology, Atomic Bomb Disease Institute, Nagasaki University Graduate School of Biomedical Sciences, 1-12-4 Sakamoto, Nagasaki, 852-8523 Japan

**Keywords:** Epidermoid cyst, Cecum, Single-incision laparoscopic surgery

## Abstract

**Background:**

Epidermoid cyst arising from the cecum is extremely rare. Single-incision laparoscopic surgery is the latest innovation in minimally invasive surgery, and shortens incisions, improves cosmesis, and reduces postoperative pain. We report here the first description of a patient with epidermoid cyst of the cecum treated by ileocecal resection by single-incision laparoscopic surgery.

**Case presentation:**

A 20-year-old woman presented to our hospital with abdominal pain in the right lower quadrant. Abdominal contrast-enhanced computed tomography showed a 56 × 35-mm cystic mass in the ileocecal area. Magnetic resonance imaging revealed a 56 × 43-mm, T1-hypointense, T2-hyperintense mass attached to the cecum. Gastrointestinal tumor or duplication cyst was suspected, and ileocecal resection was performed using single-incision laparoscopic surgery. Intraoperative examination showed the tumor as a round, whitish mass arising from the cecum. Operation time was 162 min, and intraoperative blood loss was 10 ml. Macroscopic examination showed a 56 × 45-mm elastic-hard, whitish, round mass arising from the cecal wall. Microscopic examination revealed the cyst wall lined by keratinized stratified squamous epithelium. No malignant findings were identified. The final diagnosis was epidermoid cyst of the cecum. The postoperative course was uneventful and she was discharged on postoperative day 5.

**Conclusions:**

A rare case of cecal epidermoid cyst is reported. Single-incision laparoscopic colectomy using an organ retractor represents a promising option for treating cecal epidermoid cyst.

## Background

Epidermoid cysts are generally considered to represent sequestered cystic malformations, and can develop in various parts of the body [1]. Epidermoid cysts arising from internal organs are rarely identified, but have been reported for the spleen, testes, liver and kidneys [2]. Furthermore, epidermoid cysts arising from the cecum are extremely rare [3]. Basically, epidermoid cysts is considered to have benign features, but complete tumor resection is needed due to the possibility of tumor recurrence or malignant transformation [4, 5].

Laparoscopic surgery has been the standard approach for colorectal surgery, with the benefit of better postoperative outcomes compared to an open approach [6]. Single-incision laparoscopic surgery (SILS) is the latest innovation in minimally invasive surgery, allowing shorter incisions, reduced risk of trocar-related complications, improved cosmetic outcomes, and reduced postoperative pain [7, 8].

This report offers the first description of a patient with epidermoid cyst of the cecum completely resected by ileocecal resection as an SILS.

## Case presentation

**Fig. 1:**
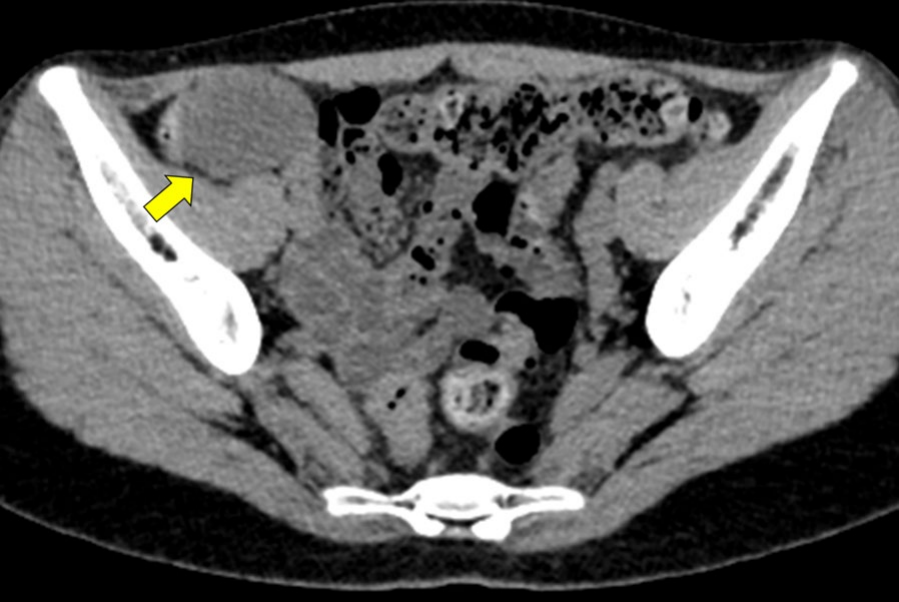
Abdominal contrast-enhanced CT. CT shows a 56 × 35-mm cystic mass with no enhancement in the ileocecal area (arrow)

**Fig. 2:**
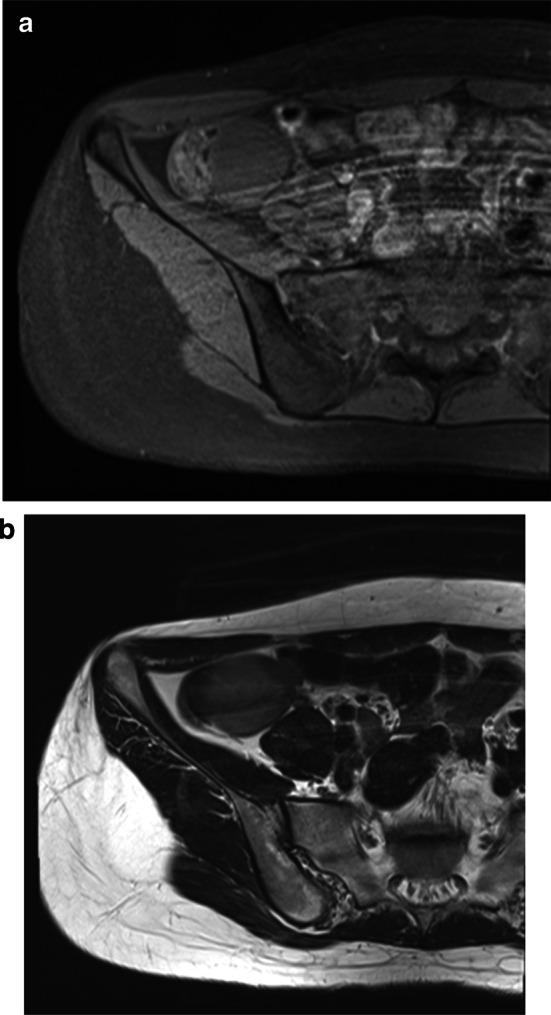
Abdominal MRI. MRI reveals a 56 × 43-mm mass attached to the cecum, appearing hypointense on T1-weighted imaging (**a**) and hyperintense on T2-weighted imaging (**b**)

A 20-year-old woman with abdominal pain in the right lower quadrant presented to hospital. Vital signs were stable. Physical examination revealed tenderness of the right lower abdomen, but she showed no muscle defense or rebound tenderness. Laboratory data showed no inflammation or anemia. Abdominal contrast-enhanced CT showed a 56 × 35-mm cystic mass with no enhancement in the ileocecal area (Fig. 1). MRI revealed a 56 × 43-mm, T1-hypointense, T2-hyperintense mass attached to the cecum (Fig. 2a, b). Gastrointestinal stromal tumor (GIST) or duplication cyst was suspected. After obtaining informed consent regarding the risk of rupture, and the possible need for an additional port or conversion to open surgery due to the risk of rupture, ileocecal resection with Japanese D3 lymph node dissection was performed as an SILS [9].

**Fig. 3:**
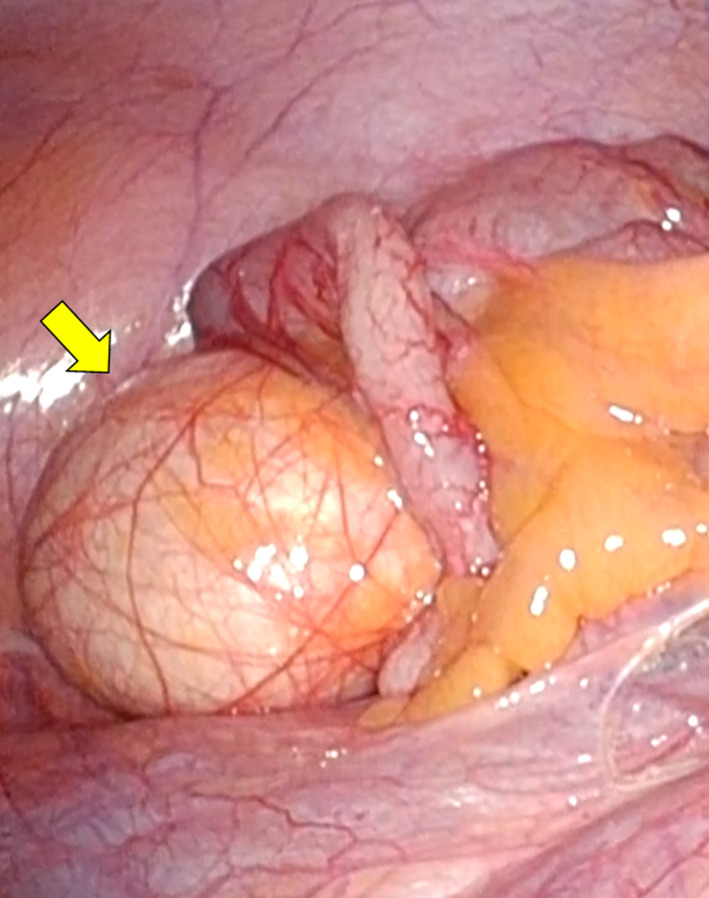
Intraoperative findings. The tumor is seen as a round, whitish mass arising from the cecum (arrow)

A 4-cm zig-zag incision was placed in the umbilicus. An EZ access (Hakko-medical, Tokyo, Japan) was then inserted, and two 5-mm ports and one 12-mm port were placed (one for the scope, two for the energy device). The tumor was seen as a round, whitish mass arising from the cecum (Fig. 3). Mobilization was started from the right-side colon using a medial approach. The pedicle of the ileocecal artery and vein was grasped by an organ retractor (B. Braun, Tokyo, Japan) to maintain a good operative field, as previously reported [10]. After the ileocecal artery and vein were transected, mobilization of the colon was completed by dissection of the lateral attachment. The lesion was removed from the body via the umbilicus. Functional end-to-end anastomosis was performed using a Signia purple 60 stapling system (Covidien, Minneapolis, MN). Operation time was 162 min and intraoperative blood loss was 10 ml.

**Fig. 4:**
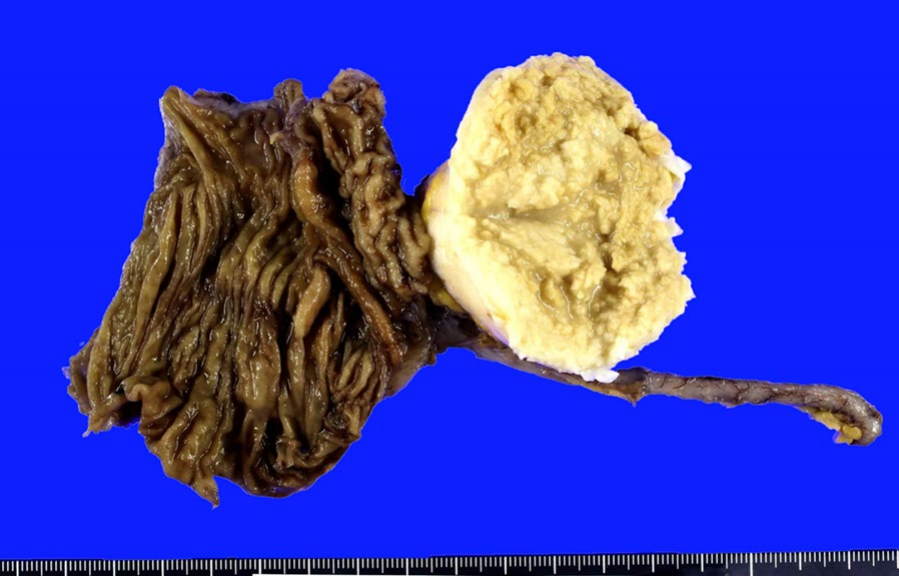
Macroscopic examination. The tumor is a 56 × 45-mm, elastic-hard, whitish, round mass arising from the cecal wall

Macroscopic examination showed a 56 × 45-mm elastic-hard, whitish, round mass arising from the cecal wall (Fig. 4).

**Fig. 5:**
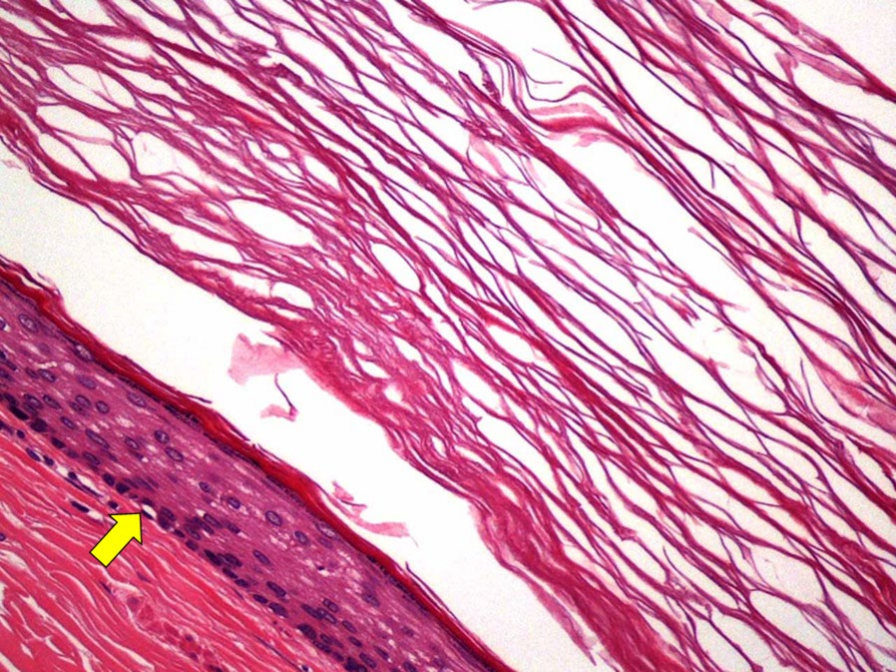
Histological examination (hematoxylin and eosin stain, ×20). The cyst wall of the tumor is lined by keratinized stratified squamous epithelium (arrow). No malignant findings are present

Microscopic examination revealed the cyst wall was lined with keratinized stratified squamous epithelium. No malignant findings were apparent (Fig. 5).

The final diagnosis was epidermoid cyst of the cecum. The postoperative course was uneventful and the patient was discharged on postoperative day 5. As of 6 months postoperatively, no evidence of recurrence has been identified.

## Discussion

The present case report describes a rare case of epidermoid cyst of the cecum treated by SILS ileocecal resection.

Cecal epidermoid cyst is extremely rare, with only 9 other cases reported in the English literature [3]. Epidermoid cyst is considered to represent a sequestered cyst with a congenital or acquired origin. Congenital epidermoid cyst is attributed to inclusion of ectodermal tissue when epithelial surfaces coalesce. Conversely, acquired epidermoid cyst is thought to develop in patients with chronic inflammation or a history of abdominal trauma and might be due to implantation of epidermis in a location favorable to growth [1]. Park et al. reported 9 cases of cecum epidermoid cyst, of which 3 cases (33.3%) involved a history of abdominal surgery [3]. We speculate that the present case involved congenital epidermoid cyst, because the patient had no history of abdominal surgery, trauma, or chronic inflammation and was aware of right lower quadrant pain for a long period of time.

Preoperative diagnosis of cecal epidermoid cyst is difficult, and no previous reports have described reach accurate preoperative diagnosis. On CT, epidermoid cyst appears as a well-demarcated, low-density mass with enhancement of the capsule following contrast administration [11]. On MRI, the tumor appears hypointense on T1-weighted imaging and hyperintense on T2-weighted imaging. However, these findings are not specific to epidermoid cyst. The cyst may also be confused with GIST and other intra-abdominal cystic lesions such as lymphatic cyst, appendiceal mucocele, mesenteric cyst, or duplication cyst [2, 12]. In the present case, CT showed a cystic mass with no enhancement and MRI revealed a T1-hyperintense, T2-hypointense mass with features similar to previous reports of epidermoid cyst. However, we suspected GIST or duplication cyst preoperatively.

Epidermoid cyst needs to be treated by complete removal of the tumor. One reason is that residual tissue and cyst wall may lead to recurrence [5]. The recurrence rate has been reported as 2% [5]. Another reason is that occasional cases have malignant potential [13]. Yang et al. revealed malignant potential in one of 60 patients (1.7%) with epidermoid cyst. A review of the Japanese literature showed that among 101 patients with presacral epidermoid cyst, six cases had squamous cell carcinoma in the cyst wall [13]. In the present case, preoperative diagnosis was difficult and we performed ileocecal resection for complete resection as in cases of malignancy.

Laparoscopic surgery has recently gained popularity as an approach to colon disease, due to better short-term outcomes including reduced blood loss, better recovery of bowel function, and shorter duration of hospitalization [6]. Epidermoid cyst of the cecum is considered to meet the indications for laparoscopic surgery as a basically benign tumor. In the English literature for cecal epidermoid cysts, only one recent case was treated by laparoscopic surgery, and the laparoscopic approach was described as beneficial for both diagnosis and treatment [12].

Several randomized controlled studies have shown that SILS offers a feasible method with better short-term outcomes compared to conventional laparoscopic surgery [8, 14–16]. However, SILS remains technically challenging due to the in-line view, instrument crowding, and loss of triangulation necessitating a high level of technical competence [14, 17]. Basically, we have to select safer procedures such as conventional laparoscopic surgery or SILS plus one port surgery when the tumor shows malignant potential. We previously performed SILS using an organ retractor to overcome the restrictions related to single-port surgery (18). This instrument can grasp and release in various positions to provide an adequate view, which could be instructive for non-expert surgeons when considering how to create a better surgical view, as in the case of SILS with an additional port. In the present case, the tumor was able to be completely resected using this procedure. However, we have to carefully consider the indications if patients display several factors that prevent performance of an SILS approach, such as bulky tumor, severe obesity, or severe adhesions, and a safer approach must be selected.

## Conclusion

In the present case, we successfully resected epidermoid of the cecum by SILS using an organ retractor. Taking into account the condition of the patient, tumor size, and malignant potential, this approach may hold promise as an option for epidermoid cyst arising from the colon.

## Abbreviations

SILS: Single-incision laparoscopic surgery
CT: Computed tomography
GIST: Gastrointestinal tumor
